# Trabeculectomy With Antimetabolite Agents for Normal Tension Glaucoma: A Systematic Review and Meta-Analysis

**DOI:** 10.3389/fmed.2022.932232

**Published:** 2022-06-28

**Authors:** Chin Lai, Shih-Chieh Shao, Yi-Hung Chen, Yu-Kai Kuo, Chi-Chun Lai, Lan-Hsin Chuang

**Affiliations:** ^1^School of Medicine, College of Medicine, Kaohsiung Medical University, Kaohsiung, Taiwan; ^2^Department of Pharmacy, Chang Gung Memorial Hospital, Keelung, Taiwan; ^3^Department of Ophthalmology, Chang Gung Memorial Hospital, Keelung, Taiwan; ^4^College of Medicine, Chang Gung University, Taoyuan, Taiwan

**Keywords:** trabeculectomy, normal tension glaucoma, systematic review, meta-analysis, mean deviation slope

## Abstract

**Background:**

Evidence regarding the impact on visual field (VF), intraocular pressure (IOP), and antiglaucoma medications from trabeculectomy with antimetabolites for normal tension glaucoma (NTG) is conflicting because of insufficient study sample sizes. The aim of this study is to systematically assess VF progression rate, IOP control and antiglaucoma medication use after trabeculectomy with antimetabolites for progressing NTG.

**Methods:**

We searched published articles on PubMed, EMBASE, and the Cochrane Central Register of Controlled Trials from database inception to March 21, 2022. We selected studies that reported VF data before and after trabeculectomy with antimetabolite agents for NTG. We followed the Preferred Reporting Items for Systematic Reviews and Meta-analyses reporting guidelines. Data were extracted by 2 independent reviewers, and a random-effects model was employed for the meta-analysis. Study outcomes were VF progression rates measured using the pooled mean deviation (MD) slope, changes in antiglaucoma medications, and IOP. Subgroup analyses of the MD slope according to mean age (over or under 65 years), baseline MD (over or under –12 dB), and baseline IOP (over or under 15 mmHg) were performed to determine the results’ robustness.

**Results:**

We included 7 retrospective observational studies (Japan: 6 studies, United States: 1 study) comprising a total of 166 eyes. Mean preoperative VF MD slopes ranged from –0.52 to –1.05 dB/year. The meta-analysis demonstrated significant MD slope improvement after trabeculectomy (pooled mean difference: 0.54 dB/year, 95% CI: 0.40 to 0.67, I^2^ = 9%). Mean age, baseline MD, and baseline IOP subgroup analyses revealed MD slope results were consistent with those of the main analyses. The mean IOP (pooled mean difference: –5.54 mmHg, 95% CI: –6.02 to –5.06, I^2^ = 0%) and mean number of antiglaucoma medications (pooled mean difference: –1.75, 95% CI: –2.97 to –0.53, I^2^ = 98%) significantly decreased after trabeculectomy. The most frequently reported early complications after trabeculectomy were hypotony, hyphema, and shallow anterior chamber.

**Conclusion:**

This systematic review and meta-analysis indicated that trabeculectomy with antimetabolites is beneficial for progressing NTG; it preserves visual function by alleviating the MD slope and reducing antiglaucoma medication use. However, several post-trabeculectomy complications should be monitored.

## Introduction

Glaucoma, the second leading cause of blindness worldwide for adults aged older than 50, (1) is characterized by retinal ganglion cell apoptosis with specific glaucomatous optic neuropathy with or without elevated intraocular pressure (IOP). Primary open-angle glaucoma (POAG) is the predominant subtype of glaucoma affecting 1.4% of Caucasians, 5.2% of those of African descent and 2.2% of south-east Asian populations aged over 60 years old. (2) Normal tension glaucoma (NTG), a form of open-angle glaucoma, accounts for up to 92% PAOG cases in Asian countries, (3–7) compared to about 31% in the United States. (8) NTG is defined as IOP consistently lower than 21 mmHg and generally presents with progressive optic nerve damage with unknown patho-mechanisms and visual field (VF) impairment (9).

The current standard treatment for glaucoma consists of IOP reduction, including medical and laser treatment, and surgical interventions. Clinical trials involving NTG have demonstrated that 30% IOP reduction is beneficial for VF progression, in comparison to untreated controls. (10, 11) Anti-glaucoma medications, such as topical beta blockers and prostaglandin analogues (PGA), are mainstream treatment options for managing NTG in clinical practice (12–15). However, if NTG progresses despite topical medical treatment, other advanced interventions, such as filtration surgery, aka trabeculectomy, may be recommended to achieve the IOP reduction target.

Trabeculectomy surgery with the wound healing modulating agents, anti-metabolites like 5-Fluorouracil (5-FU) and Mitomycin C (MMC), remains the established and most performed fistulizing procedure for NTG refractory to medical treatment. (16–18) However, with the reduction of IOP to single digits, the efficacy of VF progression rates with MD slope following trabeculectomy with antimetabolite agents on NTG differs. For example, the mean difference of MD slope was 0.8 dB/year in the Iverson et al. study with nine participants, and 0.18 dB/year in the Nakajima et al. study with 28 participants. (17, 19) Given that previous individual studies usually included small sample sizes leading to inconsistent findings, (17, 19–24) it would be important to conduct a systematic review and meta-analysis to summarize the overall operative effects, especially as regards VF progression rates, IOP, and the number of antiglaucoma medication changes, after trabeculectomy with an antimetabolite in patients with progressing NTG.

## Methods

This study followed the Preferred Reporting Items for Systematic Reviews and Meta-analyses (PRISMA) checklist (Supplementary Table 1) and the Meta-Analysis of Observational Studies in Epidemiology guidelines. Two authors (C Lai and YH Chen) independently performed study selection, data extraction, and risk-of-bias assessments. Another senior author (Shao SC) resolved any disagreements. The study protocol has been registered on PROSPERO (CRD42021281699).

### Literature Search

We searched PubMed, EMBASE, and the Cochrane Central Register of Controlled Trials for relevant studies published from inception to March 21, 2022, by using the free-text keywords of normal tension glaucoma and trabeculectomy with appropriate MeSH terms and abbreviations. The search strategy is presented in Supplementary Table 2.

### Study Selection

We selected the studies using the following inclusion criteria: (1) The studies were observational studies (including prospective or retrospective case series or cohorts) or randomized controlled trials (RCTs). (2) They included participants with a diagnosis of NTG. (3) They included participants who had received trabeculectomy with antimetabolite agents, including MMC, applied during operation or with 5-FU as an adjunct. Finally, since the preoperative MD slope may vary among NTG patients, we included only studies with pre- and postoperative MD slope outcome data to appropriately reflect the treatment effects.

We excluded the following studies: (1) Other types of publications (e.g., conference abstracts, case reports, reviews, editorials, guidelines, trial registrations, or viewpoint papers). (2) Those not specifically focused on participants with NTG subtypes. (3) Non-English-language articles. For studies with overlapping populations from the same study source (e.g., the same hospitals), we excluded the study with the shorter follow-up period.

### Data Extraction and Risk-of-Bias Assessment

We extracted study data, including study characteristics (e.g., first author name, study design, publication year, study country, sample size, mean age, and follow-up time length), visual characteristics (e.g., baseline MD, MD slope prior to and after trabeculectomy, and IOP prior to and after trabeculectomy), and treatment characteristics (e.g., the mean number of antiglaucoma medications prior to and after trabeculectomy and complications prior to and after trabeculectomy). For studies that reported visual outcome data at different follow-up times, we extracted only the data with the longest follow-up as the post-trabeculectomy data. The risk of bias for the included studies was assessed using the Quality Assessment Tool for Before-After (Pre–Post) Studies with No Control Group developed by the National Institutes of Health (25).

### Statistical Analysis

We conducted meta-analyses using Review Manager Version 5.4 (26) to investigate the changes in the MD slope before and after trabeculectomy with antimetabolite drugs. We also measured the changes in IOP and the number of antiglaucoma drugs if the data were available. We used a random-effects model in our meta-analysis because we anticipated clinical heterogeneity within the included studies. We used the I^2^ value to quantify the statistical heterogeneity across the studies. To investigate the predisposing factors that affect the treatment effects of trabeculectomy with antimetabolite agents, we conducted several subgroup analyses, including age group (mean ages older or younger than 65 years) and baseline disease severity (pre-trabeculectomy MD: over or under –12 dB (27) and pre-trabeculectomy IOP: over or under 15 mmHg). We also summarized the reported complications after trabeculectomy through descriptive analyses.

## Results

### Characteristics and Risk of Bias in Included Studies

**FIGURE 1:**
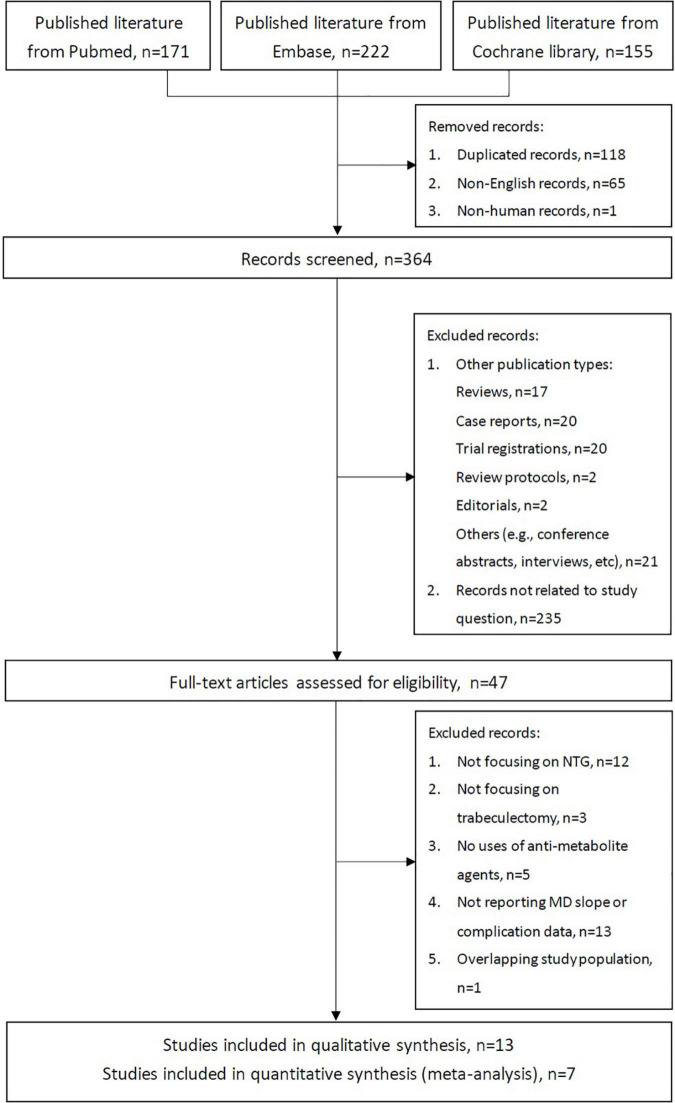
PRISMA flowchart.

The PRISMA flowchart for the study is presented in Figure 1. We initially identified 548 records from our search of three electronic biographic databases. Overall, only seven were retrospective studies, which included a total of 166 subjects from Japan (six studies, 151 eyes) and the United States (1 study, 15 eyes). These seven studies were included in this meta-analysis based on the predefined inclusion and exclusion criteria. Among these seven studies, five used MMC with a concentration of 0.2 to 0.5 mg/ml and exposure time of 1 to 5 min. (17, 19, 20, 22, 23) The other two studies used MMC at 0.4 mg/ml with exposure time of 3 to 5 min, or adjunctive use of 5-fluorouracil. (21, 24) The post-trabeculectomy follow-up periods in the studies ranged from 4.51 to 15.6 years. The details of the included studies, participants, and visual and treatment characteristics are listed in Table 1. The risk-of-bias assessment for the studies is presented in Supplementary Table 3.

**TABLE 1:** Characteristics of included studies.

Study	Region	Study design	Sample size [N(eyes)]	Age (years, mean ± SD)	Baseline MD (dB, mean ± SD)	Pre-op MD slope (follow-up) [dB/year, mean (years)]	Post-op MD slope (follow-up) [dB/year, mean (years)]	Pre-op IOP (mmHg, mean ± SD)	Post-op IOP (mmHg, mean ± SD)	IOP reduction	Pre-op medication number	Post-op medication number
Shigeeda et al. (24)	Japan	Retrospective	23	62.8 ± 10.3	N/A	−1.05 (N/A)	−0.44 (6.0)	16.2 ± 1.8	6.0 ± 1.7 (mean follow-up time: 3 years)	33%	N/A	N/A
Mataki et al. (21)	Japan	Retrospective	34	57.7 ± 9.6	−12.7 ± 5.5	−0.7 (4.6)	−0.25 (5.7)	15.7 ± 1.7	10.3 ± 2.7 (mean follow-up time: 5.7 ± 1.2 years)	34%	N/A	N/A
Naito et al. (22)	Japan	Retrospective	17	69.5 ± 7.6	−18.9 ± 4.2	−0.91 (4.3)	−0.32 (5.0)	13.9 ± 0.9	8.1 ± 2.9 (mean follow-up time: 5.0 ± 1.3 years)	42%	3.0 ± 0.4	0.8 ± 1.5
Nakajima et al. (19)	Japan	Retrospective	28	57.9 ± 10.1	−13.7 ± 7.2	−0.52 (6.3)	−0.34 (6.0)	13.9 ± 3.1	9.0 ± 4.6 (mean follow-up time: 6 years)	35%	2.6 ± 0.5	0.1 ± 0.4
Iverson et al. (17)	United States	Retrospective	15	71.8 ± 7.5	−9.5 ± 6.5	−1.05 (7.6)	−0.25 (5.4)	13.1 ± 1.5	8.5 ± 3.5 (mean follow-up time: 5.7 ± 2.3 years)	35%	2.5 ± 1.3	0.8 ± 1.3
Oie et al. (23)	Japan	Retrospective	17	55.8 ± 7.9	−11.8 ± 5.0	−0.86 (5.9)	−0.19 (15.6)	14.7 ± 1.3 (mean at follow-up)	9.1 ± 2.0 (mean follow-up time: 15.6 ± 3.2 years)	38%	0.9 ± 0.3	0.3 ± 0.4
Daugeliene et al. (20)	Japan	Retrospective	32	56.9	N/A	−0.97 (3.8)	−0.32 (4.5)	14.7 ± 1.6	8.7 ± 2.4 (mean follow-up time: 4.5 ± 1.1 years)	41%	1.5 ± 0.7	N/A

*MD, mean deviation.*

*IOP, Intraocular pressure.*

*N/A, not available.*

### Visual Field

**FIGURE 2:**
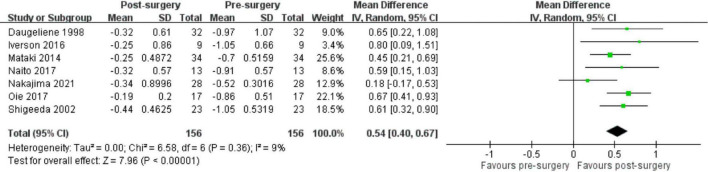
VF progression with MD slope in patients with NTG after trabeculectomy.

**FIGURE 3:**
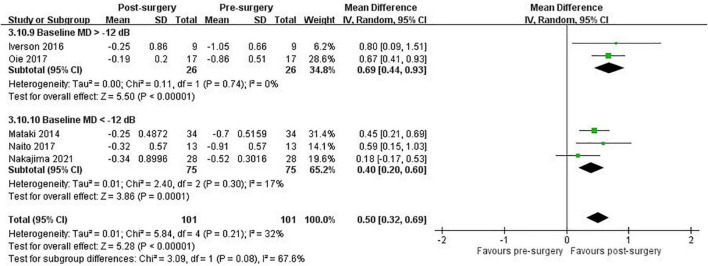
Subgroup analysis: VF progression with MD slope in patients with NTG after trabeculectomy, stratified by baseline MD group.

After data extraction in the included studies with complete MD slope data of the VF examination, the meta-analysis of seven retrospective studies comprising 156 eyes demonstrated a significant improvement in the MD slope after the patients underwent trabeculectomy, with small statistical heterogeneity (pooled mean difference: 0.54 dB/year, 95% CI: 0.40 to 0.67, I^2^ = 9%; Figure 2). Subgroup analyses indicated that the MD slope improved in different age (pooled mean difference in younger than 65 years: 0.52 dB/year, 95% CI: 0.35 to 0.68, I^2^ = 33%; pooled mean difference in older than 65 years: 0.65 dB/year, 95% CI: 0.28 to 1.02, I^2^ = 0%; test for subgroup differences: *P* = 0.53; Supplementary Figure 1), baseline MD (pooled mean difference in under -12 dB: 0.40 dB/year, 95% CI: 0.20 to 0.60, I^2^ = 17%; pooled mean difference in over –12 dB: 0.69 dB/year, 95% CI: 0.44 to 0.93, I^2^ = 0%; test for subgroup differences: *P* = 0.08; Figure 3), and baseline IOP groups (pooled mean difference in under 15 mmHg: 0.55 dB/year, 95% CI: 0.33 to 0.76, I^2^ = 31%; pooled mean difference in over 15 mmHg: 0.52 dB/year, 95% CI: 0.33 to 0.70, I^2^ = 0%; test for subgroup differences: *P* = 0.82; Supplementary Figure 2).

### IOP

**FIGURE 4:**
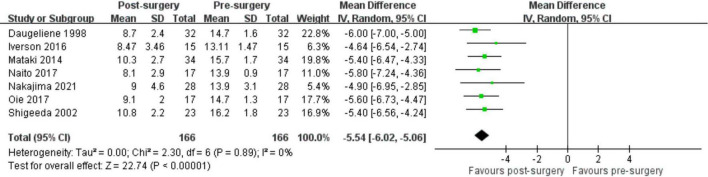
Mean change in IOP in patients with NTG before and after trabeculectomy.

The meta-analysis of seven retrospective studies comprising 166 eyes indicated a significant decrease in IOP with no statistical heterogeneity after trabeculectomy (pooled mean difference: –5.54 mmHg, 95% CI: –6.02 to –5.06, I^2^ = 0%; Figure 4). The subgroup analysis results for IOP changes according to age, baseline MD, and baseline IOP were consistent with those of the main analyses (Supplementary Figures 3–5).

### Number of Antiglaucoma Medications

After data extraction in the included studies with complete antiglaucoma medication records, the meta-analysis of 4 retrospective studies comprising 77 eyes indicated a significant decrease in the mean number of antiglaucoma medications with high statistical heterogeneity after trabeculectomy (pooled mean difference –1.75, 95% CI: –2.97 to –0.53, I^2^ = 98%; Supplementary Figure 6).

### Trabeculectomy Complications in NTG

After data extraction in the included studies with reported surgical complications after antimetabolite-adjunctive trabeculectomy, we included 7 studies (330 patients with NTG) for qualitative synthesis in this systematic review. (16, 18, 22, 28–31) Early complications after trabeculectomy, i.e., hypotony (2–48%), (18, 28, 30) hyphema (1–20%), (18, 28, 30) and shallow anterior chamber (1–16%) (28–30) were frequently reported. Among late complications, hypotonous maculopathy (3–18%) (16, 18, 22, 29–31) and bleb leak (1–19%) (16, 18, 22, 28–30) were frequently reported. Laser suturelysis, bleb needling or glaucoma eyedrops were applied in 5 studies and conservative management for other complications was noted during the follow-up period. (17, 19–21, 24) The details of the reported complications are listed in Supplementary Table 4.

## Discussion

In this systemic review and meta-analysis from seven observational studies, our findings indicated that trabeculectomy with antimetabolite drugs could significantly improve the MD slope in patients with progressing NTG. Subgroup analyses of age, baseline IOP, and baseline MD groups demonstrated results consistent with those of the main analysis and no significant differences between subgroups. Furthermore, IOP and the mean number of medications significantly decreased after trabeculectomy. Because trabeculectomy with antimetabolite drugs is a mainstream treatment strategy for patients with progressing NTG, our findings could reconfirm the treatment benefits of this procedure.

Previous studies have demonstrated the natural course of VF progression in NTG by reporting the mean MD slope, with slopes ranging from –0.33 to –0.41 dB/year. (32–34) If NTG patients are treated with adequate anti-glaucoma medications, VF progression could supposedly be alleviated, according to the findings of RCTs (12, 13, 15). However, one cohort study from De Moraes et al. demonstrated that 31% of their participants had VF progression with a slope more than −1.0 dB/year after receiving drug treatment. (13) Therefore, advanced interventions may be required to further lower IOP and prevent VF progression in progressing NTG patients. Our systematic review and meta-analysis concluded that trabeculectomy with antimetabolite drugs effectively slows VF progression, measured through the MD slope (pooled mean difference: 0.54 dB/year) in NTG patients with progressing MD slopes (-0.52 to –1.05 dB/year). This finding is especially notable because of the consistent treatment benefits across age, baseline IOP, and baseline MD groups. Although the differences of MD slope between the baseline MD groups (baseline MD under –12 dB: 0.40 dB/year and baseline MD over –12 dB: 0.69 dB/year) did not reach statistical significance (test for subgroup differences: *P* = 0.08) probably due to the suboptimal statistical power, our results may suggest a trend of patients with more favorable baseline VF conditions demonstrating more surgery-related improvement in MD slopes. The detailed mechanisms to explain these findings remain unclear, so future prospective studies are required to verify our observations.

The IOP-lowering effects of trabeculectomy with antimetabolite drugs in patients with progressing NTG have previously been reported. (16–24, 28–31) Consistent with the results of these studies, our findings quantified the mean reduction of IOP as about 5.54 mmHg after trabeculectomy, which may also explain a significant decrease in the use of antiglaucoma medications after trabeculectomy. Taking together these findings, we suggested that trabeculectomy with antimetabolite drugs was beneficial for the IOP modulation of progressing NTG. In addition to the MD slope improvement attributed to effective IOP controls after trabeculectomy with antimetabolite drugs in NTG, our findings support that low tolerance of IOP may be one patho-mechanism of NTG; (9) that is, reaching the targeted IOP reductions could prevent the visual function deterioration in NTG. However, more aggressive IOP-lowering effects following trabeculectomy with antimetabolite drugs potentially cause other ocular complications, so ophthalmologists may be very cautious when considering surgeries for progressing NTG. Although the reported complications from the included studies largely varied, we found early hypotony and late hypotonous maculopathy may occur in 2–48% and 3–18% of NTG patients after trabeculectomy, respectively. These complications may be attributed to the use of antimetabolites during ocular surgery, and careful observation and supportive treatments are often sufficient. (35) Frequent follow-up for close monitoring and timely management of possible ocular complications in progressing NTG patients after trabeculectomy with antimetabolite drugs are suggested (28).

To the best of our knowledge, this is the first systematic review and meta-analysis to evaluate the treatment effects before and after trabeculectomy with antimetabolite drugs in patients with progressing NTG. By utilizing the designs of systematic review and meta-analysis, we were able to summarize the current evidence from seven observational studies to prove the beneficial effects of this surgery on progressing NTG. This has previously remained inconsistent, probably due to insufficient sample sizes within the different individual studies. However, we must acknowledge several limitations in the present study. First, we did not identify RCTs on this topic; potential residual confounders may affect the treatment effects of trabeculectomy with antimetabolite drugs. Second, both 24–2 and 30–2 programs were used to evaluate VF progression in the included studies, but the measurement variability between these 2 programs was minor, based on a previous report. (36) Third, we found 2 included studies did not report the details on topical antiglaucoma medication use before and after trabeculectomy, which may affect the comprehensive evaluation of the surgery effects. Fourth, the clinical heterogeneity among the included studies, such the various surgical techniques of trabeculectomy, should be noted before interpreting our findings. Fifth, we excluded non-English-language articles from this systematic review, but these excluded studies were mostly from Japan. Because we included several Japanese studies published in English in this meta-analysis, we considered the potential influence of language restrictions on our findings to be minor. Finally, most of the included studies were from Japan, so we suggested further studies from other countries to confirm our findings.

This systematic review and meta-analysis suggested that trabeculectomy with antimetabolite drugs may significantly improve VF by alleviating the MD slope in progressing NTG, regardless of age, baseline IOP, and baseline MD. This surgery could also lower IOP and reduce antiglaucoma drug use. However, several surgery complications, such as early hypotony and late hypotonous maculopathy, should be clinically monitored.

## Data Availability Statement

The original contributions presented in this study are included in the article/Supplementary Material, further inquiries can be directed to the corresponding author/s.

## Author Contributions

CL and L-HC: concept and design, full access to all the data in the study, and responsibility for the data integrity and analysis accuracy. CL, S-CS, Y-HC, and L-HC: acquisition, analysis, or interpretation of the data. CL, S-CS, and L-HC: drafting of the manuscript. CL: statistical analysis. L-HC: supervision. All authors contributed in critical revision of the manuscript for important intellectual content.

## Conflict of Interest

The authors declare that the research was conducted in the absence of any commercial or financial relationships that could be construed as a potential conflict of interest.

## Publisher’s Note

All claims expressed in this article are solely those of the authors and do not necessarily represent those of their affiliated organizations, or those of the publisher, the editors and the reviewers. Any product that may be evaluated in this article, or claim that may be made by its manufacturer, is not guaranteed or endorsed by the publisher.

## Supplementary Material

The Supplementary Material for this article can be found online at: https://www.frontiersin.org/articles/10.3389/fmed.2022.932232/full#supplementary-material
