# Supplementary material for: Trabeculectomy With Antimetabolite Agents for Normal Tension Glaucoma: A Systematic Review and Meta-Analysis
Source: Front Med (Lausanne). 2022 Jun 28;9:932232. doi: 10.3389/fmed.2022.932232 (PMC9273818; doi:10.3389/fmed.2022.932232)
Supplement: Supplementary file 1 [file Data_Sheet_1.DOCX]

Supplementary Material

| **Supplementary Table 1: PRISMA checklist** | | | |
| --- | --- | --- | --- |
| **Section/topic** | **#** | **Checklist item** | **Reported on page #** |
| **TITLE** | | |  |
| Title | 1 | Identify the report as a systematic review, meta-analysis, or both. | 1 |
| **ABSTRACT** | | |  |
| Structured summary | 2 | Provide a structured summary including, as applicable: background; objectives; data sources; study eligibility criteria, participants, and interventions; study appraisal and synthesis methods; results; limitations; conclusions and implications of key findings; systematic review registration number. | 1-2 |
| **INTRODUCTION** | | |  |
| Rationale | 3 | Describe the rationale for the review in the context of what is already known. | 2 |
| Objectives | 4 | Provide an explicit statement of questions being addressed with reference to participants, interventions, comparisons, outcomes, and study design (PICOS). | 2 |
| **METHODS** | | |  |
| Protocol and registration | 5 | Indicate if a review protocol exists, if and where it can be accessed (e.g., Web address), and, if available, provide registration information including registration number. | 3 |
| Eligibility criteria | 6 | Specify study characteristics (e.g., PICOS, length of follow-up) and report characteristics (e.g., years considered, language, publication status) used as criteria for eligibility, giving rationale. | 3 |
| Information sources | 7 | Describe all information sources (e.g., databases with dates of coverage, contact with study authors to identify additional studies) in the search and date last searched. | 3 |
| Search | 8 | Present full electronic search strategy for at least one database, including any limits used, such that it could be repeated. | Supplementary Table 2 |
| Study selection | 9 | State the process for selecting studies (i.e., screening, eligibility, included in systematic review, and, if applicable, included in the meta-analysis). | Figure 1 |
| Data collection process | 10 | Describe method of data extraction from reports (e.g., piloted forms, independently, in duplicate) and any processes for obtaining and confirming data from investigators. | 3 |
| Data items | 11 | List and define all variables for which data were sought (e.g., PICOS, funding sources) and any assumptions and simplifications made. | 3 |
| Risk of bias in individual studies | 12 | Describe methods used for assessing risk of bias of individual studies (including specification of whether this was done at the study or outcome level), and how this information is to be used in any data synthesis. | 3 |
| Summary measures | 13 | State the principal summary measures (e.g., risk ratio, difference in means). | 3-4 |
| Synthesis of results | 14 | Describe the methods of handling data and combining results of studies, if done, including measures of consistency (e.g., I^2^) for each meta-analysis. | 3-4 |

| **Section/topic** | **#** | **Checklist item** | **Reported on page #** |
| --- | --- | --- | --- |
| Risk of bias across studies | 15 | Specify any assessment of risk of bias that may affect the cumulative evidence (e.g., publication bias, selective reporting within studies). | N/A |
| Additional analyses | 16 | Describe methods of additional analyses (e.g., sensitivity or subgroup analyses, meta-regression), if done, indicating which were pre-specified. | 4 |
| **RESULTS** | | |  |
| Study selection | 17 | Give numbers of studies screened, assessed for eligibility, and included in the review, with reasons for exclusions at each stage, ideally with a flow diagram. | Figure 1 |
| Study characteristics | 18 | For each study, present characteristics for which data were extracted (e.g., study size, PICOS, follow-up period) and provide the citations. | Table 1 |
| Risk of bias within studies | 19 | Present data on risk of bias of each study and, if available, any outcome level assessment (see item 12). | Supplementary Table 3 |
| Results of individual studies | 20 | For all outcomes considered (benefits or harms), present, for each study: (a) simple summary data for each intervention group (b) effect estimates and confidence intervals, ideally with a forest plot. | Figure 2-4 |
| Synthesis of results | 21 | Present results of each meta-analysis done, including confidence intervals and measures of consistency. | Figure 2-4 |
| Risk of bias across studies | 22 | Present results of any assessment of risk of bias across studies (see Item 15). | N/A |
| Additional analysis | 23 | Give results of additional analyses, if done (e.g., sensitivity or subgroup analyses, meta-regression [see Item 16]). | Supplementary Figure 1-6 |
| **DISCUSSION** | | |  |
| Summary of evidence | 24 | Summarize the main findings including the strength of evidence for each main outcome; consider their relevance to key groups (e.g., healthcare providers, users, and policy makers). | 5 |
| Limitations | 25 | Discuss limitations at study and outcome level (e.g., risk of bias), and at review-level (e.g., incomplete retrieval of identified research, reporting bias). | 6 |
| Conclusions | 26 | Provide a general interpretation of the results in the context of other evidence, and implications for future research. | 7 |
| **FUNDING** | | |  |
| Funding | 27 | Describe sources of funding for the systematic review and other support (e.g., supply of data); role of funders for the systematic review. | 7 |

**Supplementary Table 2: Search Strategy**

| **PubMed** |
| --- |
| 1. (“Trabeculectomy”[Mesh]) OR (trabeculectomy) OR (“trabeculectomy”) OR (“filtration surgery”) OR (filtration surgery) 2. (“Low Tension Glaucoma”[Mesh]) OR (“low tension Glaucoma”) OR (“low pressure glaucoma”) OR (“normal pressure glaucoma”) OR (“normal tension glaucoma”) OR (“sine tension glaucoma”) 3. #1 AND #2 |
| **Embase** |
| 1. “trabeculectomy” OR “filtration surgery” 2. “normal tension glaucoma” OR “low tension glaucoma” OR “normal pressure glaucoma” OR “low pressure glaucoma” OR “sine tension glaucoma” 3. #1 AND #2 AND　[study type: human; publication type: article] |
| **Cochrane Central Register of Controlled Trials** |
| 1. MeSH descriptor: [Trabeculectomy] explode all trees 2. (trabeculectomy) 3. (filtration surgery) 4. MeSH descriptor: [Low Tension Glaucoma] explode all trees 5. (low tension glaucoma) OR (normal tension glaucoma) OR (normal pressure glaucoma) OR (low pressure glaucoma) OR (sine tension glaucoma) 6. (#1 OR #2 OR #3) AND (#4 OR #5) |

**Supplementary Table 3: Methodological qualities of included studies determined through Quality Assessment Tool for Before-After (Pre-Post) Studies With No Control Group (National Institute of Health’s Systematic Evidence Reviews and Clinical Practice Guidelines)**

| Author, Year | Quality criteria item | | | | | | | | | | | | Quality Rating |
| --- | --- | --- | --- | --- | --- | --- | --- | --- | --- | --- | --- | --- | --- |
|  | Item 1 | Item 2 | Item 3 | Item 4 | Item 5 | Item 6 | Item 7 | Item 8 | Item 9 | Item 10 | Item 11 | Item 12 |  |
| Daugeliene et al., 1998 | Yes | Yes | CD | Yes | CD | Yes | Yes | CD | Yes | Yes | Yes | NA | Fair |
| Oie et al., 2017 | Yes | Yes | CD | Yes | CD | Yes | Yes | CD | Yes | Yes | Yes | NA | Fair |
| Shigeea et al., 2002 | Yes | Yes | CD | Yes | CD | Yes | Yes | CD | Yes | Yes | Yes | NA | Fair |
| Mataki et al., 2014 | Yes | Yes | CD | Yes | CD | Yes | Yes | CD | Yes | Yes | Yes | NA | Fair |
| Naito et al., 2017 | Yes | Yes | CD | No | CD | Yes | Yes | CD | No | Yes | Yes | NA | Fair |
| Nakajima et al., 2021 | Yes | Yes | CD | Yes | CD | Yes | Yes | CD | Yes | Yes | Yes | NA | Fair |
| Iverson et al., 2016 | Yes | Yes | CD | Yes | CD | Yes | Yes | CD | No | Yes | Yes | NA | Fair |

Abbreviations: CD, cannot determine; NA, not applicable; NR, not reported

**Assessment items:**

Item 1: Was the study question or objective clearly stated?

Item 2: Were eligibility/selection criteria for the study population prespecified and clearly described?

Item 3: Were the participants in the study representative of those who would be eligible for the test/service/intervention in the general or clinical population of interest?

Item 4: Were all eligible participants that met the prespecified entry criteria enrolled?

Item 5: Was the sample size sufficiently large to provide confidence in the findings?

Item 6: Was the test/service/intervention clearly described and delivered consistently across the study population?

Item 7: Were the outcome measures prespecified, clearly defined, valid, reliable, and assessed consistently across all study participants?

Item 8: Were the people assessing the outcomes blinded to the participants’ exposures/interventions?

Item 9: Was the loss to follow-up after baseline 20% or less? Were those lost to follow-up accounted for in the analysis?

Item 10: Did the statistical methods examine changes in outcome measures from before to after the intervention? Were statistical tests done that provided p values for the pre-to-post changes?

Item 11: Were outcome measures of interest taken multiple times before the intervention and multiple times after the intervention (i.e., did they use an interrupted time-series design)?

Item 12: If the intervention was conducted at a group level (e.g., a whole hospital, a community, etc.) did the statistical analysis take into account the use of individual-level data to determine effects at the group level?

| **Supplementary Table 4: Complications after trabeculectomy with antimetabolite drugs for NTG** | | | | | | | | |
| --- | --- | --- | --- | --- | --- | --- | --- | --- |
|  | Study (sample size) | | | | | | | |
|  | Hagiwara (21) | Naito (17) | Schultz (30) | Jongsareejit (39) | Membry MMC (25) | Membry 5-FU (36) | Jayaram (131) | Yamamoto (31) |
| Early hypotony | NA | NA | **6 (20%)** | NA | **12 (48%)** | **9 (25%)** | **3 (2%)** | NA |
| Early hyphema | NA | NA | **6 (20%)** | NA | **5 (20%)** | **6 (17%)** | **1 (1%)** | NA |
| Early choroidal effusion | NA | NA | **1 (3%)** | NA | **4 (16%)** | **7 (19%)** | **0 (0%)** | NA |
| Early choroidal detachment | NA | NA | NA | **9 (23%)** | NA | NA | NA | NA |
| Early shallow anterior chamber | NA | NA | NA | **6 (15%)** | **4 (16%)** | **4 (11%)** | **1 (1%)** | NA |
| Early bleb leak | NA | NA | NA | NA | **2 (8%)** | **7 (19%)** | **1 (1%)** | NA |
| Hypotony | NA | **9 (53%)** | **9 (30%)** | NA | **7 (28%)** | NA | **1 (1%)** | NA |
| Choroidal effusion | NA | NA | **1 (3%)** | NA | NA | NA | NA | NA |
| Choroidal detachment | **1 (5%)** | **4 (24%)** | NA | NA | NA | NA | NA | **2 (6%)** |
| Hypotonous maculopathy | **1 (5%)** | **3 (18%)** | **2 (7%)** | **7 (18%)** | **3 (12%)** | **1 (3%)** | **0 (0%)** | **3 (10%)** |
| Hyphema | NA | **4 (24%)** | NA | NA | NA | NA | NA | NA |
| Shallow anterior chamber | **2 (10%)** | **2 (12%)** | NA | NA | NA | NA | NA | **2 (6%)** |
| Cataract | **6 (29%)** | NA | NA | **3 (8%)** | NA | NA | **4 (3%)** | **7 (23%)** |
| Cataract surgery | NA | NA | NA | NA | **3 (12%)** | **3 (8%)** | NA | NA |
| VA decline by ≥0.1 unit | NA | **3 (18%)** | NA | NA | NA | NA | NA | NA |
| VA decrease by 2 Snellen lines | NA | NA | NA | NA | NA | NA | NA | NA |
| Corneal epithelial defect | **1 (5%)** | NA | NA | NA | NA | NA | NA | **1 (3%)** |
| Cystoid macular edema | NA | NA | **3 (10%)** | NA | NA | NA | NA | NA |
| Persistent diplopia | NA | NA | **1 (3%)** | NA | NA | NA | NA | NA |
| Chronic or recurrent iritis | NA | NA | **1 (3%)** | NA | NA | NA | NA | NA |
| Persistent corneal edema | NA | NA | **2 (7%)** | NA | NA | NA | NA | NA |
| Dysesthesia | NA | NA | **3 (10%)** | NA | NA | NA | NA | NA |
| Bleb leak | **4 (19%)** | **1(6%)** | **2 (7%)** | **1 (3%)** | **3 (12%)** | NA | **1 (1%)** | NA |
| Blebitis | NA | NA | **1 (3%)** | **2 (5%)** | NA | NA | **0 (0%)** | NA |
| Endophthalmitis | NA | NA | NA | NA | **2 (8%)** | NA | **0 (0%)** | NA |
| Lid ptosis | NA | NA | NA | NA | NA | NA | **2 (2%)** | NA |
| NA: not reported | | | | | | | | |

**
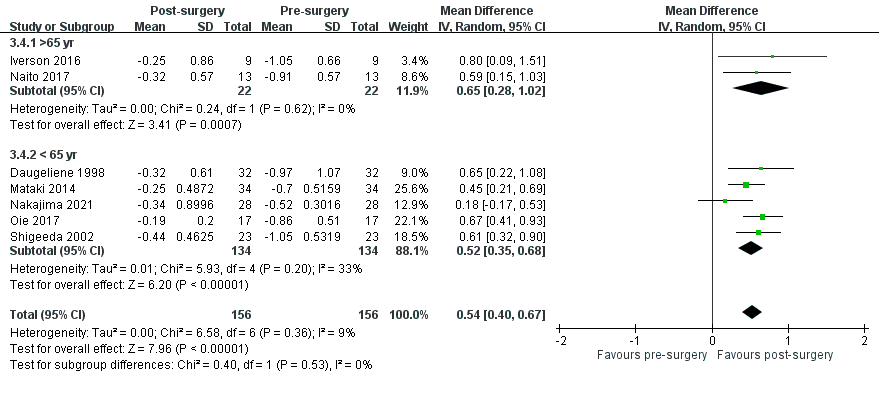
Supplementary Figure 1. Subgroup analysis: Visual field progression with MD slope in patients with NTG following trabeculectomy, stratified by age group**

**
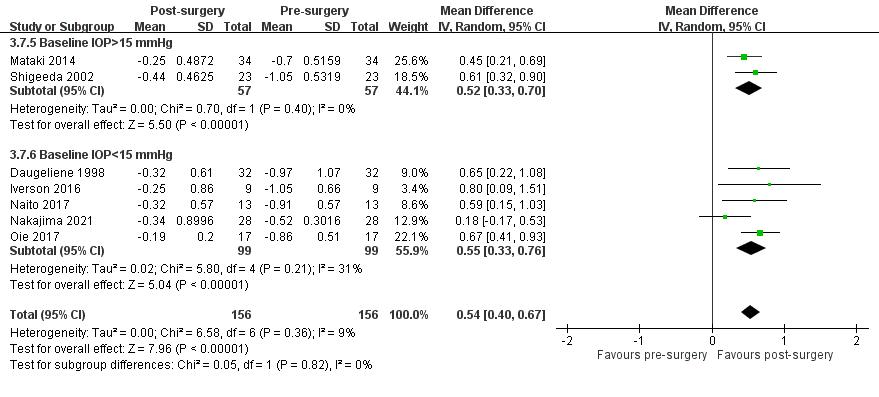
Supplementary Figure 2. Subgroup analysis: Visual field progression with MD slope in patients with NTG following trabeculectomy, stratified by baseline IOP group**

**
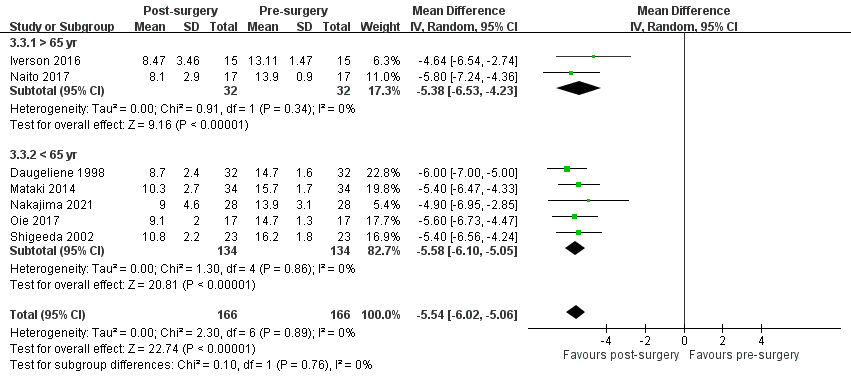
****Supplementary Figure 3. Subgroup analysis: IOP change in patients with NTG following trabeculectomy, stratified by age group**

**
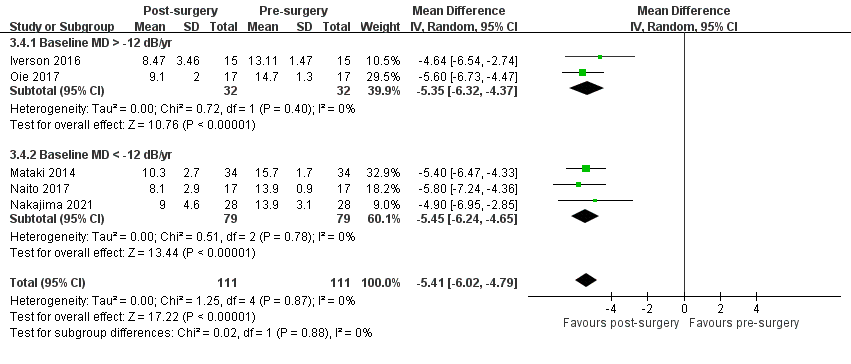
****Supplementary Figure 4. Subgroup analysis: IOP change in patients with NTG following trabeculectomy, stratified by baseline MD group**

**
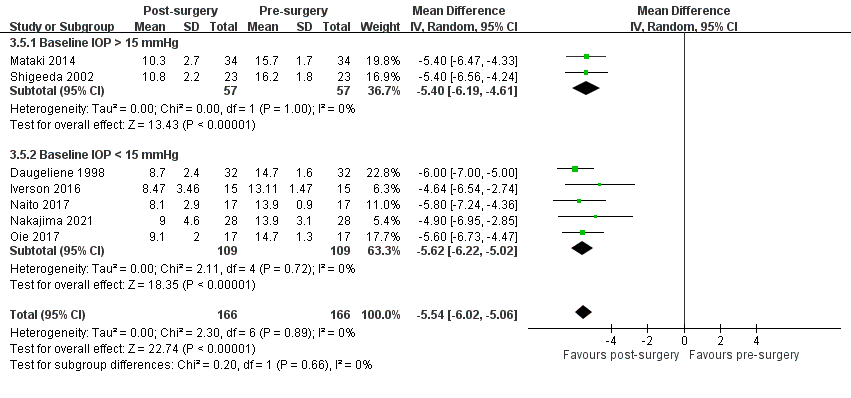
Supplementary Figure 5. Subgroup analysis: IOP change in patients with NTG following trabeculectomy, stratified by baseline IOP group**

**
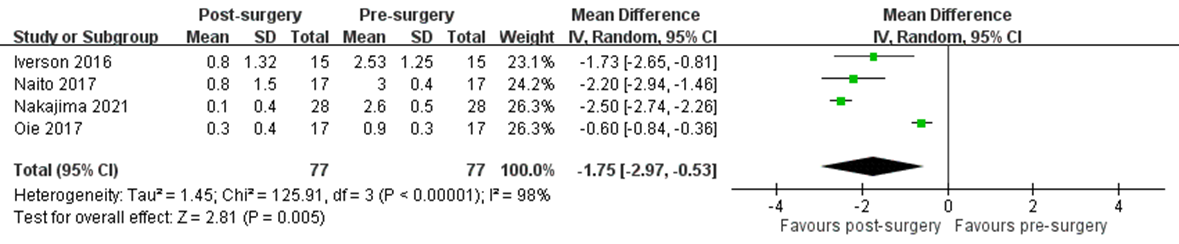
**

**Supplementary Figure 6. Mean change in number of antiglaucoma medications in patients with NTG before and after trabeculectomy**
